# Human Structural Homologues of SARS-CoV-2 PL ^pro^ as Anti-Targets: A Strategic Panel Analysis

**DOI:** 10.17912/micropub.biology.001418

**Published:** 2025-04-14

**Authors:** Abdullah I. Al-Homoudi, Joseph Engel, Michael D. Muczynski, Joseph S. Brunzelle, Navnath S. Gavande, Ladislau C. Kovari

**Affiliations:** 1 Dept. of Biochemistry, Microbiology and Immunology, Wayne State University, School of Medicine, Detroit, MI, USA; 2 Synchrotron Research Center, Life Science Collaborative Access Team, Northwestern University, Argonne, IL, USA; 3 Dept. of Pharmaceutical Sciences, Eugene Applebaum College of Pharmacy and Health Sciences, Wayne State University, Detroit, MI, USA; 4 Molecular Therapeutics Program, Barbara Ann Karmanos Cancer Institute, Wayne State University, School of Medicine, Detroit, MI, USA

## Abstract

COVID-19 is caused by SARS-CoV-2, a highly transmissible and pathogenic RNA betacoronavirus. Developing small-molecule antiviral inhibitors of the SARS-CoV-2 papain-like protease (PL ^pro^ ) is advantageous due to the enzyme's role in processing viral polyproteins and disrupting host immune sensing. Given the structural and functional similarities between PL ^pro^ and human deubiquitinases (DUBs), small-molecule inhibitors are frequently counter-screened for off-target activity using a panel of human DUBs. Through X-ray crystallography, DALI structural comparisons, and *in silico* analysis, a high-quality crystal structure of SARS-CoV-2 PL ^pro^ enabled the identification of the closest structural human homologues of PL ^pro^ . Among the 27 human DUBs identified, USP46 and USP12 displayed the greatest structural similarity to PL ^pro^ , with alignment scores below 0.45 and RMSD values of 3.0 Å or less. Additionally, binding sites on ubiquitin-specific protease (USP46) and USP12, ancillary to the active site residues, share high sequence identity to the PL ^pro^ substrate binding sites that are often engaged by the most potent PL ^pro^ inhibitors. These findings offer a strong basis for choosing anti-targets and serve as a foundation for designing selective small-molecule PL ^pro^ inhibitors.

**Figure 1. Identifying the closest structural human homologues of SARS-CoV-2 PL pro:**
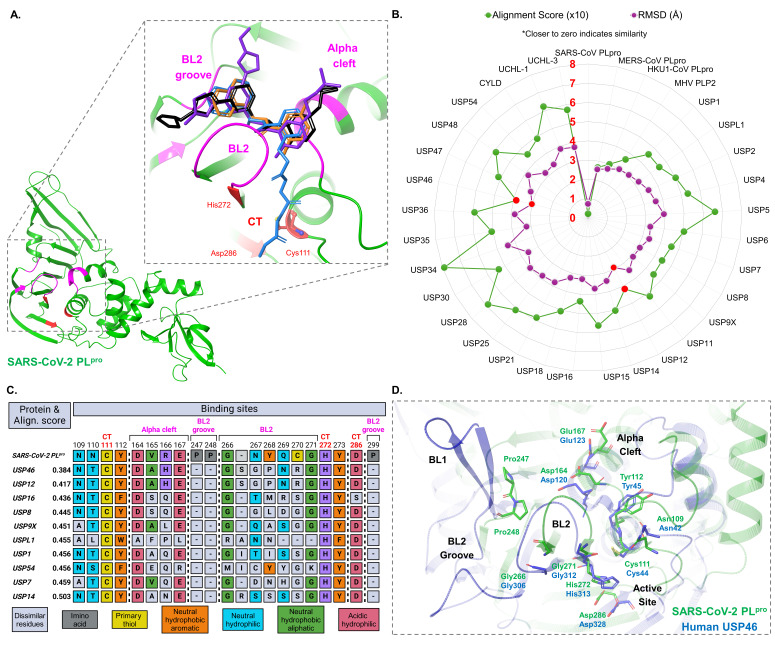
( **A** ) The structure of SARS-CoV-2 PL ^pro^ (9BF8.pdb) is represented by a green, pink, and red ribbon, alongside the binding poses of the most potent small-molecule inhibitors of SARS-CoV-2 PL ^pro^ . The inhibitors (thick tube representations) include GRL-0617 (orange, 7CJM.pdb), Compound 7 (blue, 8EUA.pdb), XR8-24 (black, 7LBS.pdb), and Jun12682 (purple, 8UOB.pdb), which interact with binding sites (pink) that are ancillary to active site residues Cys111, His272, and Asp286 (red). The relevant binding sites are known as the blocking loop (BL2) (Gly266-Gly-271), BL2 groove (Pro247, Pro248, Pro299, Gly266), and the Alpha-cleft (Asp164-Glu167) binding sites (pink). ( **B** ) Radar plot illustrating structural alignment results of related coronavirus (CoV) PL ^pro^ from SARS-CoV, MERS-CoV, HKU1-CoV, murine hepatitis virus (MHV), and the catalytic domains of 27 human deubiquitinases (DUBs). Alignment scores (green) lower than 0.6 indicate a good alignment, while scores greater than 0.6 indicate a failure of structural alignment calculation and insufficient structural similarity for a meaningful alignment. RMSD (Å) (purple) is the distance between superpositioned atoms, calculated based only on the residues that have been successfully aligned. The plot highlights the closest human structural homologues of SARS-CoV-2 PL ^pro^ , which are ubiquitin-specific protease 46 (USP46) and USP12 (red data blocks). ( **C** ) Binding site amino acid sequence alignment and Alignment scores of unrelated human structural protease homologues of SARS-CoV-2 PL ^pro^ (Created in BioRender. Alhomoudi, A. (2025) https://BioRender.com/gpsv9cp). ( **D** ) The figure illustrates the structural superposition of binding sites for USP46 (blue, 5L8H.pdb) and SARS-CoV-2 PL ^pro^ (green, 9BF8.pdb), emphasizing the BL2 groove, Alpha-cleft, and BL1, a secondary blocking loop present on USP46 in place of the BL2 groove.

## Description

Despite the global availability of SARS-CoV-2 protease inhibitor regimens, there remains a critical need to enhance their pharmacological properties, particularly in terms of oral bioavailability, metabolic stability, and specificity (Eng et al., 2022; Jiang et al., 2023; Shimizu et al., 2023). The Papain-like protease (PL ^pro^ ) is essential for the replication of human coronaviruses (hCoVs) as it processes polyproteins translated from viral RNA into functional nonstructural proteins (NSPs). In addition to processing viral polyproteins, hCoV PL ^pro^ directly facilitates the evasion of host immune responses by cleaving ubiquitin and the ubiquitin-like interferon-stimulated gene 15 (ISG-15) from host protein conjugates (Thiel et al., 2003; Barretto et al., 2005; Linder et al., 2007; Linder et al., 2005; Shin et al., 2020). Therefore, inhibiting PL ^pro^ offers a promising approach to reducing viral replication and boosting antiviral immunity.

Deubiquitination and deISGylation are essential regulatory mechanisms in many biological processes, including NF-ĸB signaling following host innate immune system activation during viral infection (Hochstrasser, 2000; Song & Li, 2021). Seven human Deubiquitinase (DUB) subfamilies function to cleave ubiquitin and ubiquitin-like adducts from substrate proteins (Balakirev et al., 2003; Burnett et al., 2003; Evans et al., 2003; Gan-Erdene et al., 2003; Verma et al., 2002; Wilkinson, 1997; Yeh et al., 2000). The ubiquitin-specific protease (USP) subfamily has raised particular interest due to its presence in different eukaryotic organisms and the varied functions ascribed to this subfamily of diverse proteases in normal and pathological conditions (Quesada et al., 2004). In 2005, around the same time that Quesada and colleagues identified 22 novel human USPs, the first crystal structure of the SARS-CoV PL ^pro^ was elucidated (2FE8.pdb) (Ratia et al., 2006). Structural similarity screening through the entire Protein Data Bank (PDB) identified two significant human structural homologues of SARS-CoV PL ^pro^ : USP14 and HAUSP7 (USP7) (Ratia et al., 2006). Since then, tens of thousands of crystal structures have been deposited into the PDB, and the emergence of the deadly MERS-CoV in 2012 and SARS-CoV-2 in 2019 has accelerated the structure-based drug design (SBDD) of protease inhibitors.

The outbreak of SARS-CoV in 2002 facilitated the development of the naphthyl methyl amine core from GRL-0617 as an inhibitor for PL ^pro^ (Ghosh et al., 2009). Since then, GRL-0617 has been repurposed against SARS-CoV-2 PL ^pro^ , and several potent analogs have been optimized using SBDD, including Compound 19, Compound 7, XR8-24, and Jun12682 (Ratia et al., 2008; Sanders et al., 2023; Shan et al., 2021; Tan et al., 2023) ( **Fig. 1A** ). These potent antiviral compounds (Enzymatic IC _50_ ≥ 0.09 μM, Antiviral EC _50_ ≥ 0.42 μM) exhibit comparable binding poses, interacting with residues involved in ubiquitin and ISG-15 recognition, as evidenced by various hCoV PL ^pro^ X-ray co-crystal structures with ubiquitin (4MM3.pdb, 4RF1.pdb, 6XAA.pdb) and ISG-15 (6XA9.pdb, 6BI8.pdb, 5TL6.pdb). Notably, the flexible “blocking loop” (BL2) (Gly266-Gly271), the hydrophobic BL2 groove (Pro247, Pro248, Pro299, Gly266), and the rigidifying Alpha cleft (Asp164-Glu167) of PL ^pro^ , which are ancillary to the catalytic triad (Cys111, His272 & Asp286), improve inhibitor potency upon engagement. Although the efficacy of these compounds has been confirmed, the primary focus is now on enhancing pharmacological and drug-like attributes, including specificity and off-target activity.

Currently, the rationale for selecting specific USP panels for *in vitro* analysis to evaluate the specificity of PL ^pro^ inhibitors and their off-target activity remains unclear. In the development of GRL-0617, Ratia et al. counter-screened against USP7, USP18, UCH-L1, and UCH-L3 (Ratia et al., 2008). In the development of Compound 19, Shan et al. counter-screened against USP36, USP14, USP8, USP7, USP2, and related DUBs UCH-L1, SNEP1, OTUB-1, ATAXIN3, and AMSH (Shan et al., 2021). Sanders et al. screened Compound 7 against USP2c, USP4, USP7, USP8, USP15, USP30, and UCH-L1 (Sanders et al., 2023). Lastly, XR8-24 and Jun12682 were counter-screened against only USP7 and USP14 (Tan et al., 2023). SBDD efforts would greatly benefit from a clearly defined panel of human DUBs, facilitating a more rational counter-screening of lead PL ^pro^ inhibitors. To systematically address this concern, we identified a panel of the closest structural human homologues of SARS-CoV-2 PL ^pro^ to counter-screen potential drug candidates

We crystallized the SARS-CoV-2 PL ^pro^ enzyme (9BF8.pdb), and this high-quality crystal structure served as the query structure. The **9BF8 PDB Validation Report** contains relevant data collection and refinement statistics. The diffraction data were collected by the Life Sciences Collaborative Access Team (LS-CAT) using the NSLS-II 17-1 AMX beamline at the Brookhaven National Laboratory, resulting in a 1.85 Å resolution data set. The resulting refined model had an overall B-factor of 34.1 Å ^2^ , allowing proper main and side chain modeling. The phased and refined structure resulted in a final *R*_work_ of 17.75% ( *R*_free_ = 20.00%), with zero Ramachandran and sidechain outliers.

A heuristic search of the PDB was performed using the DALI server (ekhidna2.biocenter.helsinki.fi/dali) to compare the coordinates of the query protein structure with all protein chains deposited in the PDB. The catalytic domains of 27 USPs were identified to have significant DALI Z-scores, where a higher score indicates statistical significance in similarity. DALI Z-scores for identified USPs ranged from 9.5 to 13.8, with most having similar scores ( **Data Set Identifying Structural Homologues of SARS-CoV-2 PL ^pro^** ). SARS-CoV-2 PL ^pro^ sequence identity of the identified USPs ranged from 9% to 16%. Other hits include SARS-CoV PL ^pro^ and related hCoV PL ^pro^ from MERS-CoV, hCoV-HKU1, and the murine hepatitis virus (MHV), which had Z-scores ranging from 32 to 44 and SARS-CoV-2 PL ^pro^ sequence identities ranging from 28-83%.

Unlike the DALI server analysis, alignment metrics, such as the Alignment score in Schrödinger Maestro Suite release 2024-2 (version 14.0.134), provide a more comprehensive evaluation, offering a detailed and holistic view of protein similarity based on sequence and structure. In combination with the 27 USP catalytic cores identified by DALI, MERS-CoV PL ^pro^ , hCoV-HKU1 PLP2, and Murine Hepatitis Virus (MHV) PLP2 were processed using the Maestro Protein Preparation Workflow to prepare structures, primarily to fill in missing side chains, and then subjected to the Protein Structure Alignment tool (Sastry et al., 2013). As expected, related CoV PL ^pro^ had the best alignment scores to SARS-CoV-2 PL ^pro^ , ranging from 0.021 to 0.3, respectively ( **Fig. 1B** ). The catalytic domains of USP46 and USP12 exhibited the highest structural homology with SARS-CoV-2 PL ^pro^ , with alignment scores below 0.6 and RMSD values of ~3 Å. The alignment scores of USP46 (0.368) and USP12 (0.384) were similar to the alignment score of the MHV PL ^pro^ (0.361), highlighting the high similarity between the viral and human proteases ( **Fig. 1C** ). The catalytic domains of USP16, USP8, USP9X, USPL1, USP1, USP54, and USP7 are also significantly close structural homologues of PL ^pro^ with alignment scores of less than 0.459 and RMSD of less than 3.37 Å. Nonetheless, USP16 lacks essential catalytic residues and is categorized as a non-protease homologue in accordance with the MEROPS database classification ( **Fig. 1C** ). This designation rules USP16 out as a viable PL ^pro^ off-target. Furthermore, USPL1 and USP54 exhibit low PL ^pro^ sequence identity in the BL2 and Alpha-cleft regions. Lastly, USP7’s active site requires ubiquitin substrate binding for the catalytic Cys to align into an active conformation, and its BL2 is in an open state when HAUSP is unbound to ubiquitin (Hu et al., 2002), rendering it an irrational anti-PL ^pro^ off-target. Overall, USP46, USP12, and USP9X are the closest human structural homologues to PL ^pro^ ( **Superposition of USP46, USP12, and USP9X to SARS-CoV-2 PL ^pro^** ) and serve as the most rational off-targets for testing the specificity of small-molecule PL ^pro^ inhibitors. Specifically, USP46 is notable for its superior alignment score, low RMSD value, and high sequence/structural identity with the ancillary binding sites of PL ^pro^ (e.g., BL2) ( **Fig. 1D** ).

The methodology described can identify anti-target panels for developing antiviral compounds. The findings presented herein provide a more transparent and economical array of human proteins suitable for counter-screening compounds. This advancement consequently facilitates the development of potent and selective antiviral small-molecule PL ^pro^ inhibitors.

## Methods

**PL ^pro^ Cloning, Expression, and Purification**

The gene encoding the full-length SARS-CoV-2 PL ^pro^ (isolate Wuhan-Hu-1 NC_045512.2, pp1ab aa 1564–1878) with N-terminal His _6_ -SUMO-cleavable tag was cloned into pMCSG7 (Midwest Center for Structural Genomics) vector following the Ligation Independent Cloning (LIC) procedure (Stols et al., 2002). Whole Plasmid Sequencing was performed by Plasmidsaurus using Oxford Nanopore Technology with custom analysis and annotation. The pHis _6_ -SUMO vector containing the PL ^pro^ gene was transformed into Rosetta2 *E. coli* cells, plated onto Amp ^+^ agar plates, and incubated overnight at 37°C. Single colonies were cultured overnight in LB media with 100 µg/mL Amp at 37°C until OD _600_ = 1.0. The temperature was then lowered to 20°C for 1 hour, followed by the induction of protein expression with 0.4 mM IPTG. The next day, cell pellets were harvested by centrifugation and frozen at -80°C. Frozen cell pellets were lysed by sonication in lysis buffer (25 mM Tris-HCl, pH 7.5, 150 mM NaCl, 1 mM DTT) containing 10 µg/mL lysozyme, 0.5 µg/mL Aprotinin, and 0.05 µg/mL Leupeptin. The lysate was clarified by centrifugation and loaded onto a 5 mL Ni-NTA resin equilibrated with Ni column wash buffer (25 mM Tris HCl, pH 7.5, 150 mM NaCl). Bound His _6_ -SUMO-PL ^pro^ was eluted with elution buffer (25 mM Tris-HCl, pH 7.5, 150 mM NaCl, and 300 mM imidazole). Fractions containing His _6_ -SUMO-PL ^pro^ were pooled and exchanged into SUMO dialysis buffer (20 mM Tris-HCl, pH 7.5, 250 mM NaCl, 1 mM DTT). A 1:100 molar ratio of ULPL1 (SUMO-specific protease) to PL ^pro^ was incubated at 4°C overnight to cleave the His _6_ -tag. The reaction was loaded onto a Ni-NTA resin re-equilibrated with 25 mM Tris-HCl, pH 7.5, and 150 mM NaCl to remove the tag and the SUMO-specific protease. Cleaved PL ^pro^ eluted first, followed by gel filtration. PL ^pro^ fractions were pooled, concentrated, and frozen in liquid nitrogen for storage at -80°C in S75 buffer (20 mM Tris-HCl, pH 7.5, 250 mM NaCl, 1 mM DTT). A Bradford assay using BSA as a standard monitored protein yield at each step.

**Crystallization and structure determination of SARS-CoV-2 PL ^pro^**

The crystals of SARS-CoV-2 PL ^pro^ were grown using the sitting drop vapor diffusion method against a 300 µL reservoir solution at 4°C. The PL ^pro^ crystals were formed by mixing 1-2 µL of 18.7 mg/mL protein with 1 µL of reservoir solution containing 0.1 M Tris buffer (pH 7.0), 1 M sodium phosphate monobasic monohydrate (NaH2PO4), 1 M potassium phosphate dibasic (K2HPO4), and 3% w/v sucrose. Crystals formed over 1-2 days, reaching dimensions of 0.5-0.8 mm with octahedral and dodecahedral external symmetries. Full-sized crystals were harvested after one week. They were carefully transferred into a cryoprotectant consisting of mother liquor and 25% (v/v) glycerol before being flash-frozen in liquid nitrogen. The samples were subsequently shipped to the NSLS-II 17-1 AMX beamline at Brookhaven National Laboratory for diffraction data collection. Reflections were integrated and scaled using XDS and AIMLESS via Xia2 (P. Evans, 2006; Kabsch, 2010; Winter, 2010). Phases were calculated through molecular replacement using PHASER within the PHENIX software package (Adams et al., 2010; Liebschner et al., 2019; McCoy et al., 2007) for molecular structure determination by employing a monomeric SARS-CoV-2 PL ^pro^ structure (8FWN.pdb) as the search model. Automated model building and multiple rounds of refinement were conducted using PHENIX Autobuild, WinCoot, and PHENIX Refine (Afonine et al., 2012; Afonine et al., 2018; Echols et al., 2012; Emsley, Lohkamp, Scott, & Cowtan, 2010; Terwilliger et al., 2008). The X-ray crystal structure of SARS-CoV-2 PL ^pro^ has been deposited in the PDB (9BF8.pdb), and the deposition validation file is also provided.

**Structure Similarity Analysis**

The query crystal structure PDB coordinates (9BF8.pdb) were uploaded into the DALI protein structure comparison server to perform a heuristic PDB search. Identified structures were prepared and aligned using the Protein Preparation Workflow and Protein Structure Alignment tool, employing the query crystal structure as reference residues using Schrödinger Maestro Suite release 2024-2 (version 14.0.134).

## Reagents

**Table:** 

**Plasmid**	**Description**
pHis _6_ -SUMO-PL ^pro^	Custom bacterial vector with an ampicillin resistance marker for regulated expression of SARS-CoV-2 PL ^pro^ with a cleavable His _6_ -SUMO tag.

## Data Availability

Description: Table with DALI-Z and Schrödinger Maestro Suite metrics used for analysis. . Resource Type: Dataset. DOI: https://doi.org/10.22002/cq58r-x9918 Description: Full wwPDB X-ray structure validation report and statistics for PDB code 9BF8 SARS-CoV-2 PLpro Untagged Crystal Structure.. Resource Type: Collection. DOI: https://doi.org/10.22002/ncxnv-byp09 Description: Structural superposition of USP46, USP12, and USP9X to SARS-CoV-2 PLpro highlighting relevant binding sites.. Resource Type: Image. DOI: https://doi.org/10.22002/7dxa1-p2863
